# Trehalose Protects Keratinocytes against Ultraviolet B Radiation by Activating Autophagy via Regulating TIMP3 and ATG9A

**DOI:** 10.1155/2022/9366494

**Published:** 2022-04-12

**Authors:** Li Li, Hongying Chen, Xu Chen, Sihan Chen, Heng Gu

**Affiliations:** ^1^Central Laboratory, Institute of Dermatology, Chinese Academy of Medical Science & Peking Union Medical College, Nanjing 210042, China; ^2^Department of Physical Therapy, Institute of Dermatology, Chinese Academy of Medical Science & Peking Union Medical College, Nanjing 210042, China; ^3^Jiangsu Key Laboratory of Molecular Biology for Skin Diseases and STIs, Institute of Dermatology, Chinese Academy of Medical Science & Peking Union Medical College, Nanjing 210042, China

## Abstract

Trehalose, a natural disaccharide, is synthesized by many organisms when cells are exposed to stressful stimuli. On the basis of its ability to modulate autophagy, trehalose has been considered an innovative drug for ameliorating many diseases, but its molecular mechanism is not well described. Previous findings demonstrated that trehalose plays a photoprotective role against ultraviolet (UV) B-induced damage through autophagy induction in keratinocytes. In this study, coimmunoprecipitation, label-free quantitative proteomic and parallel reaction monitoring, and western blot analysis demonstrated that trehalose promotes the interaction between tissue inhibitor of metalloproteinase (TIMP) 3 and Beclin1. Western blot and immunofluorescence staining analysis suggested that trehalose increases ATG9A localization in lysosomes and decreases its localization in the endoplasmic reticulum. Furthermore, in the presence or absence of UVB radiation, we evaluated the influence of TIMP3 and ATG9A small interfering RNA (siRNA) on the effect of trehalose on autophagy, cell death, migration, or interleukin-8 expression in keratinocytes, including HaCaT, A431, and human epidermal keratinocytes. The results revealed that in HaCaT cells, TIMP3 and ATG9A siRNA resulted in attenuation of trehalose-induced autophagy and inhibited cell death. In A431 cells, TIMP3 and ATG9A siRNA led to attenuation of trehalose-induced autophagy and cell death and inhibited migration. In human epidermal keratinocytes, trehalose-induced autophagy and inhibition of the interleukin-8 expression were blocked by ATG9A but not TIMP3 siRNA. In addition, the results of quantitative real-time PCR and immunohistochemistry analysis demonstrated the abnormal expression of TIMP3 and ATG9A in actinic keratosis and cutaneous squamous cell carcinoma skin tissues. These findings suggest the protective effects of trehalose in normal keratinocytes and its inhibitory effects on cancerous keratinocytes, possibly mediated by activation of autophagy and regulation of TIMP3 and ATG9A, providing the mechanistic basis for the potential use of trehalose in the prevention or treatment of UVB-induced skin diseases.

## 1. Introduction

Trehalose, a natural disaccharide, is synthesized by many organisms, not including vertebrates, when cells are exposed to stressful stimuli, including dehydration, heat, oxidation, hypoxia, or even anoxia. Increasing evidence demonstrates the bioprotective and low toxicity properties of trehalose, indicating its utility in humans, including in the food, cosmetic, and pharmaceutical industries [1]. Recently, trehalose has been proposed as a potential preventative treatment for SARS-CoV-2 infection and transmission [2]. In the past, as a disaccharide, trehalose was considered to be impermeable to cell membranes. However, trehalose has since been revealed to pass into the cytosol, affecting intracellular signaling and organelle functions [3]. In the presence of a high extracellular concentration, trehalose is taken up and conveyed to the lysosomal pathway [1, 4]. Due to its high chemical stability, trehalose is not degraded but leaks into the cytoplasm. In addition, trehalose exerts protective cellular effects by rescuing mitochondrial dysfunction and suppressing endoplasmic reticulum (ER) stress [5].

Autophagy is an intracellular catabolic mechanism through which surplus cellular compartments are recycled and damaged organelles are destroyed to protect against cellular stresses. The autophagy process is initiated by the sequestration of cytoplasmic components in double-membrane autophagosomes that transport substrates to lysosomes for degradation [6]. Autophagy is evolutionarily conserved, with more than 40 autophagy-related genes (ATGs) identified in yeast, many of which have human orthologs [7]. The ULK complex with ATG1, ATG13, ATG17, and ATG9, phosphatidylinositol 3-kinase catalytic subunit type (PI3KC) 3 complex with Beclin1 and ATG5-ATG12-ATG16 complex are three important complexes that play key roles in the initiation and elongation steps of autophagic vesicle formation. In our previous study, we demonstrated that trehalose induces autophagy through a mechanistic target of rapamycin- (mTOR-) independent pathway characterized by unchanged ATG protein levels [8].

Many studies have led to the hypothesis that trehalose promotes aggregate clearance and exhibits a protective effect by regulating inflammation, proliferation, and apoptosis by modulating autophagy. For example, in human corneal cells, trehalose induces autophagy and reduces stress-induced secretion of the cytokines interleukin- (IL-) 6, IL-8, and monocyte chemoattractant protein-1 [9]. In vascular smooth muscle cells, trehalose inhibits cell proliferation by activating transcription factor EB, a master regulator of autophagy signaling [10]. In human podocytes, trehalose decreases apoptosis by inducing autophagy [11].

Human skin, the largest organ of the body, serves as a barrier against environmental factors, among which solar ultraviolet (UV) radiation is an important physical mutagenic and cancerogenic factor. Exposure to UV radiation is a major cause of oxidative stress, resulting in differential expression of endogenous antioxidant enzymes, protein oxidation, and lipid peroxidation, consequently leading to skin inflammation, DNA damage, immunosuppression, and tumorigenesis [12]. UV radiation that reaches the surface of the earth is divided into UVA (315-400 nm) and UVB (280-315 nm) based on wavelength [13]. Although comprising merely 5% of UV radiation, UVB is considered to play a key role in the occurrence and development of many skin diseases, including actinic keratoses (AK) and cutaneous squamous cell carcinoma (cSCC), as it is more energetic than UVA [14]. The protective effect of trehalose against UVB-induced photodamage in human keratinocytes (HaCaT cells) and the anti-inflammatory, antiapoptotic, and particularly antioxidant properties of trehalose in UVB-irradiated corneas have been previously reported [15]. Expression levels of the autophagy molecular marker microtubule-associated protein 1 light chain LC (LC) 3 (a homolog of yeast ATG8)-II differ in AK and cSCC tissues [16]. However, until now, few studies have described the underlying mechanism of autophagy responsible for the protective effect of trehalose against UVB radiation or related skin diseases.

In this study, we identified that the interaction between tissue inhibitor of metalloproteinase (TIMP) 3 and Beclin1 and the translocation of ATG9A from the ER to lysosomes is promoted by trehalose. In the presence and absence of UVB radiation, we evaluated the influence of TIMP3 and ATG9A small interfering RNA (siRNA) on the effect of trehalose on autophagy, cell death, migration, and IL8 expression in keratinocytes, including HaCaT, A431, and human epidermal keratinocytes (HEKs). In addition, the mRNA and protein levels of TIMP3 and ATG9A were assayed in AK and cSCC skin tissues.

## 2. Materials and Methods

### 2.1. Reagents and Antibodies

Reagents used in this study include chloroquine diphosphate (Life technologies, P36235), D-(+)-trehalose dehydrate (Sigma-Aldrich, T0167), acridine orange (AO) (Sigma-Aldrich, A9231), calcein AM (Dojindo Laboratories, C326), LysoTracker Deep Red (ThermoFisher Scientific, L12492), Mito-Tracker Green (Beyotime Biotechnology, C1048), ER-Tracker Red (Beyotime Biotechnology, C1041), DAPI Staining Solution (Beyotime Biotechnology, C1005), and TRIzol Reagent (Invitrogen, 15596026). For 100 mM concentration, 5.67 g trehalose is dissolved to 10 mL phosphate-buffered saline (PBS).

Primary antibodies used in western blot: LC3A/B (12741), ATG14 (96752), Rubicon (8465), PI3KC3 (4263), PIK3R4 (14580), UVRAG (13115), Bcl2 (2870), GAPDH (5174), PARP (9542), caspase-3 (9665), RPA32 (RPA2) (mouse, 52448), ATR (2790), phospho-ATR Ser428 (2853), ATM (2873), phospho-ATM Ser1981 (5883), phospho-Chk1 Ser345 (2348), phospho-histone H2A.X Ser139 (9718), ATG5 (8540), ATG9A (13509), COX IV (4850), IRE1*α* (3294), and histone H3 (4499), these antibodies are purchased from Cell Signaling Technology. Beclin1 (ab207612), ATG3 (ab108251), TIMP3 (ab39184), MMP9 (ab76003), and ATG7 (ab133528), these antibodies are purchased from Abcam. Beclin1 (Cell Signaling Technology, 4112) was used in coimmunoprecipitation (co-IP). ATG5 (Abcam, ab108327) was specially used in colocalization, ATG9A (Abcam, ab108338) was specially used in immunofluorescence (IF) staining and immunohistochemistry (IHC) analysis and TIMP3 (Proteintech, 10858-1-AP) was specially used in IHC analysis.

### 2.2. Cell Culture

HaCaT (a cell line of non-tumorigenic human keratinocytes) and A431 (a cell line of cSCC) were cultured in Dulbecco's Modified Eagle's Medium (Gibco, 11965-092) supplemented with 10% fetal bovine serum. HEKs were obtained from neonatal foreskin as described previously [17] and cultured in defined keratinocyte serum free media (Gibco, 10785-012). All cells were maintained in a humidified atmosphere of 5% CO_2_ at 37°C.

### 2.3. Human Skin Specimens

The skin tissue was obtained from and deidentified by Jiangsu Biobank of Clinical Resources. Informed consent was obtained from all patients who contributed samples in this study.

### 2.4. UVB Irradiation

The cells at 80-90% confluence were washed with PBS, then covered with a small amount of PBS and exposed to UVB radiation (50 mJ/cm^2^) by UVB lamps (Philips UVB Broadband PL-S 9 W/12) for 34 seconds. UV light was between 290 and 320 nm with a peak at 310 nm. The irradiance was 1.50 mW/cm^2^ at a distance of 16 cm.

### 2.5. Western Blot Analysis

Total cellular protein extraction: cells were lysed by RIPA lysis buffer (Beyotime Biotechnology, P0013C) containing phosphatase and protease inhibitors (Roche Applied Science) on ice for 10 minutes, and the cell debris was removed by centrifugation at 12,000 rpm 4°C for 5 minutes.

Mitochondrial and cytoplasmic protein was extracted by Cell Mitochondria Isolation Kit (Beyotime Biotechnology, C3601). Cells (5 × 10^7^) were collected by centrifugation after trypsinization and then lysed by 1 mL mitochondria isolation reagent containing 10 *μ*L PMSF at 4°C for 10 minutes. After vortex for 30 seconds, the cell debris was removed by centrifugation at 1,000 g 4°C for 10 minutes. Mitochondria (precipitation) and cytoplasmic protein (supernatant) in supernatant were separated by centrifugation at 3,500 g 4°C for 10 minutes. Mitochondria (precipitation) was lysed by 100 *μ*L mitochondria lysis buffer containing 1 *μ*L PMSF at 4°C for 10 minutes, and the debris was removed by centrifugation at 12,000 g 4°C for 10 minutes to obtain mitochondrial protein. Meanwhile, the cytoplasmic protein (supernatant) was collected by centrifugation at 12,000 g 4°C for 10 minutes.

ER protein was extracted by ER Protein Isolation Kit (Bestbio, BB-314541) according to the manufacture's introduction.

Nuclear and cytoplasmic protein was extracted by NE-PER Nuclear and Cytoplasmic Extraction Reagents (ThermoFisher Scientific, 78833) according to the manufacturer's introduction.

Protein concentrations were determined using a BCA Protein Assay Kit (Beyotime Biotechnology, P0010). Equal amounts of protein (20 *μ*g) were loaded onto 4-15% precast polyacrylamide gels (Bio-Rad Laboratories, 4561083) and then transferred onto polyvinylidene fluoride membranes (Bio-Rad Laboratories, 1620177). The membrane was blocked with 3% BSA in tris-buffered saline with tween-20 (TBST). After blocking, the membrane was incubated with primary antibodies (1 : 1000) at 4°C overnight, washed with TBST, and incubated with HRP-labeled goat anti-rabbit secondary antibody (1 : 2000) (Cell Signaling Technology, 7074). The protein bands were visualized using Clarity™ Western ECL Substrate (Bio-Rad Laboratories, 170-5061). Density of protein bands were quantified by Quantity One software. COX IV (mitochondrial protein), GAPDH (total and cytoplasmic protein), histone H3 (nuclear protein), and IRE1*α* (ER protein) were served as loading controls.

### 2.6. Cell Proliferation Assay

Bromo-deoxyuridine (BrdU) incorporation into replicating DNA is a commonly used and highly reproducible method to monitor cell proliferation. CytoSelect™ BrdU Cell Proliferation ELISA Kit (Cell Biolabs, CBA-251) and Cell Counting Kit-8 (CCK-8) (Beyotime Biotechnology, C0038) were used according to the manufacturer's introduction. Cells were seeded in 48-well plates at a density of 1.5 × 10^5^ cells/well and incubated for 24 hours before siRNA, trehalose, or UVB exposure treatment. In BrdU assay, first, cells were incubated with BrdU (10 *μ*M) for 4 hours. Second, cells were washed with PBS and fixed with fix/denature solution at 37°C for 30 minutes. Third, cells were incubated with the antibody diluent, anti-BrdU antibody, and secondary antibody HRP conjugate at room temperature for 1 hour in order. Finally, substrate solution was added and incubated at room temperature for 15 minutes, and stop solution was added to stop the enzyme reaction. BrdU incorporation was determined by measuring the optical density value (OD) at wavelength 370 nm and 492 nm and calculated as OD 370 nm-OD 492 nm. In CCK-8 assay, 20 *μ*L CCK-8 solution was added and incubated at 37°C for 2 hours. The OD 450 nm was measured.

### 2.7. Apoptosis Assay

Cell apoptosis analysis was performed using fluorescein isothiocyanate (FITC) Annexin V Apoptosis Detection Kit I (BD Biosciences, 556547). Cells were collected by 0.25% trypsin without EDTA and resuspended at 5 × 10^6^ cells/mL in 100 *μ*L binding buffer. Cells were stained with 5 *μ*L Annexin V-FITC and 5 *μ*L propidium iodide (PI) for 20 minutes in the dark. Before detection, cells were resuspended to 500 *μ*L volume. Cell apoptosis was assayed using a flow cytometer (BD FACS Verse). Data was analyzed using FlowJo software.

### 2.8. Migration Measurement

Cells were plated into Radius™ 96-Well Cell Migration Assay plate (Cell Biolabs, CBA-126) at a density of 5 × 10^4^ cells/well. Each plate well contains a 0.68 mm circular nontoxic, biocompatible hydrogel spot where cells cannot attach. At the start of the experiment, the stoppers were removed (the cells could populate the circular void space), and the culture medium was replaced with fresh one in the presence or absence of trehalose. After incubation for indicated time, cells were stained with 5 *μ*M calcein AM, and images of the gap closure were acquired using a digital camera attached to inverted microscope. The circular space was quantitatively evaluated by Quantity One software. The cell migration rate was calculated by the following formula: (100% − areas without cell migration/area isolated by stopper) × 100.

### 2.9. Lactate Dehydrogease (LDH) Release Assay

LDH release was detected using a LDH Cytotoxicity Assay Kit (Beyotime, C0017) according to the manufacturer's introduction to evaluate cell death. In brief, cells were plated into 24-well plates and incubated for at least 24 hours before trehalose, UVB exposure, or siRNA treatment. LDH release was determined by measuring the OD 490 nm and OD 600 nm and calculated as OD 490 nm-OD 600 nm. The percentage of cell death was calculated as (△OD of cell culture supernatants of trehalose, UVB, or siRNA treated group–△OD of cell culture supernatants of control group)/(△OD of cell culture supernatants of maximum LDH release group–△OD of cell culture supernatants of control group) × 100.

### 2.10. Co-IP

Cells were lysed by Cell Lysis Buffer for Western and IP (Beyotime Biotechnology, P0013) containing phosphatase and protease inhibitors. The lysate was incubated with anti-Beclin1 antibody (1 : 50) and rotated gently overnight at 4°C. The mixture was then incubated with SureBeads™ Protein G Magnetic beads (Bio-Rad Laboratories, 161-4023) at 4°C for 4 hours. For western blot analysis, beads were washed with PBST (PBS with tween-20) and resuspended in boiling 1× SDS sample buffer. For mass spectrometry analysis, 100 *μ*L Pierce™ IgG Elution Buffer pH 2.0 (ThermoFisher Scientific, 21028) was added to beads and vortex at 37°C for 10 minutes. Repeat the elution process and collect the eluted proteins. Finally, 20 *μ*L 1 M Tris-HCl pH 8.8 was added (Beyotime Biotechnology, ST788) for neutralization.

### 2.11. Cell Transfection and RNA Interference

Specific siRNA (100 nM) transfection was performed with Lipofectamine 2000 (Invitrogen, 11668-019) according to the manufacturer's instruction. After transfection for at least 48 hours, cells were treated with trehalose, UVB, or both for indicated time. These are the sequences of siRNA used in this study: siTIMP3: 5′-GCA GUA CAU CCA UAC GGA ATT-3′ (sense), 5′-UUC CGU AUG GAU GUA CUG CTT-3′ (antisense). siATG9A: 5′-CCU CAA GGC CGA GUA CAA ATT-3′ (sense), 5′-UUU GUA CUC GGC CUU GAG GTT-3′ (antisense). Negative control (NC): 5′-UUC UCC GAA CGU GUC ACG UTT-3′ (sense), 5′-ACG UGA CAC GUU CGG AGA ATT-3′ (antisense).

### 2.12. Label-Free Quantitative Proteomic and Parallel Reaction Monitoring (PRM) Analysis

Proteins interacted with Beclin1 were enriched by co-IP. Label free quantitative proteomic and PRM analysis was by performed by Aksomics Bio-tech (Shanghai, China). Peptides were analyzed on a *Q* Exactive mass spectrometer (Thermo Fisher Scientific) connected to the Nano-UPLC Liquid Chromatography system EASY-nLC1200 (Thermo Fisher Scientific). The full mass and subsequent MS/MS analysis was performed with the resolution at M/Z 200 of 70,000 for MS1 and 35,000 for MS2, respectively. The automatic gain control for MS1 and MS2 was at 1*E*+6 and 1*E*+5, respectively. The maximum ion injection time for MS1 and MS2 was 100 ms and 120 ms, respectively. Normalized collision energy was at 28%, isolation window was at 2.0 m/z, and time of dynamic exclusion was at 30 seconds. Raw data were processed with MaxQuant (1.6.1.0). ComplexHeatmap and Volcano plot were performed to show differently expressed proteins. For PRM, after nanoUPLC separation and MS/MS, the PRM data was imported into Skyline for transition extraction.

### 2.13. IF Staining Assay

Cultured cells were fixed with 4% paraformaldehyde for 30 minutes and then permeabilized in 0.1% Triton X-100 for 10 minutes. After blocking with 3% BSA in TBST for 1 hour, cells were incubated with primary antibodies (anit-ATG3 (1 : 200), anti-ATG5 (1 : 200), anti-ATG7 (1 : 200), anti-ATG9A (1 : 100), and anti-LC3A/B (1 : 200)) at 4°C overnight and then secondary antibodies (1 : 1000) (Cell Signaling Technology, 4412, AlexaFluor 488 anti-rabbit IgG, 4414, AlexaFluor 647 anti-rabbit IgG,) for 2 hours at room temperature. LysoTracker™ Deep Red, Mito-Tracker Green, ER-Tracker Red, and DAPI were used to visualize the lysosome, mitochondrion, ER, and nuclei according to the manufacturer's introduction, respectively. Cells were observed and photographed with a laser confocal microscope (Olympus Corporation, FV1000). Cells with emerged bright yellow were identified as positive cells. Colocolization analysis of positive cells was performed by ImageJ software, and Pearson's *R* value (above threshold) was determined.

### 2.14. AO Staining Assay

Cells were incubated with 5 *μ*g/mL AO at 37°C for 10 minutes. Cells were observed and photographed with a laser confocal microscope (Olympus Corporation, FV1000). The excitation wavelength was set at 488 nm, and the emission wavelength was set at 515 nm to obtain green fluorescence; the excitation wavelength was set at 546 nm, and the emission wavelength was set at 620 nm to obtain red fluorescence. Red and green fluorescence intensities in cells were determined by Quantity One software.

### 2.15. RFP-GFP-LC3B Puncta Analysis

RFP-GFP-LC3B was transfected into cells using Premo™ Tandem Autophagy Sensor RFP-LC3B-GFP Kit (Life technologies, P36239) according to the manufacturer's introduction. Upon induction of autophagy and fusion of lysosomes, the pH drops, the acid-sensitive GFP (green) quenches, and the acid-insensitive RFP (red) or merged puncta (yellow) appears. Cells were transfected for 36 hours and then irradiated by UVB or treated with trehalose. Cells were observed and photographed with a laser confocal microscope (Olympus Corporation, FV1000). The excitation wavelength was set at 405 nm, the emission wavelength was set at 461 nm to obtain blue fluorescence (DAPI), the excitation wavelength was set at 488 nm, the emission wavelength was set at 520 nm to obtain green fluorescence (GFP), the excitation wavelength was set at 543 nm, and the emission wavelength was set at 618 nm to obtain red fluorescence (RFP). Cells with yellow puncta were considered as positive.

### 2.16. Quantitative Real-Time PCR (RT-qPCR)

The total RNA of skin tissues and cells was isolated with TRIzol Reagent. cDNA was synthesized from 500 ng total RNA by PrimeScript™ RT Master Mix (Takara, RR036A). RT-qPCR analysis was conducted using the ABI 7300 Real-Time PCR System (Perkin-Elmer Applied Biosystems) with iTaq Universal SYBR Green Supermix (Beyotime Biotechnology, 172512). The relative quantification of mRNA levels was normalized to U6 or GAPDH. The primers were as follows: U6, forward: 5′-GCT TCG GCA GCA CAT ATA CTA AAA T-3′, reverse: 3′-CGC TTC ACG AAT TTG CGT GTC AT-5′; GAPDH, forward: 5′-CAG TGC CAG CCT CGT CTA T-3′, reverse: 3′-AGG GGC CAT CCA CAG TCT TC-5′; TIMP3, forward: 5′-GGT CTA CAC CAT CAA GCA GAT GAA G-3′, reverse: 5′-AGG CCA CAG AGA CTC TCG GAA G-3′; ATG9A, forward: 5′-TCC AGT ACA AGG CAG TGT TCA-3′, reverse: 5′-CGA ACA TCC ATC TGA GCA AAG G-3′; IL8, forward: 5′-TCC AAG CTG GCC GTG GCT CT-3′, reverse: 5′-CTG TGT TGG CGC AGT GTG GTC C-3′.

### 2.17. IHC Analysis

Tissue sections were deparaffinized and subjected to antigen retrieval. Slides were blocked with 3% BSA in PBS for 30 minutes, then incubated with primary antibodies (anti-ATG9A (1 : 50) and anti-TIMP3 (1 : 200)) at 4°C overnight and then HRP-labeled goat anti-rabbit secondary antibody (1 : 200) (SeraCare, 5220-0336) at room temperature for 1 hour. Subsequently, the slides were stained and visualized with DAB, counterstained with hematoxylin, washed with 1% hydrochloric acid solution, dehydrated, and stabilized with mounting medium. Two visions of every slide were chosen and photographed, and the integrated optical density was determined by Image-Pro Plus 6.0 software.

### 2.18. Enzyme-Linked Immunosorbent Assay (ELISA)

The ELISA assay was used to quantify the levels of secreted IL8 protein using a Human IL-8 ELISA Kit (BD Bioscences, 550999) according to the manufacturer's introduction.

### 2.19. Statistical Analysis

The results were evaluated by the GraphPad Prism software, and data were analyzed using ANOVA. The results are presented as means ± standard deviation (SD), and all results are the mean of at least three independent experiments. Multiple comparisons between the groups were performed using Tukey test. Statistical significance was set at ^∗∗∗^*P* values (*P*) ≤ 0.001, ^∗∗^*P* ≤ 0.01, and ^∗^*P* ≤ 0.05.

## 3. Results

### 3.1. Pretreatment with Trehalose Induces Autophagy and Inhibits the Migration of HaCaT Cells

In our previous study, trehalose was demonstrated to induce autophagic flux in HaCaT cells and to protect cells from UVB radiation [8]. However, the time effect of trehalose on UVB-irradiated keratinocytes was not evaluated in detail. To our knowledge, UVB damage to keratinocytes should be healed over time, and the curative effect is based on when and how the drug is delivered.

**Figure 1:**
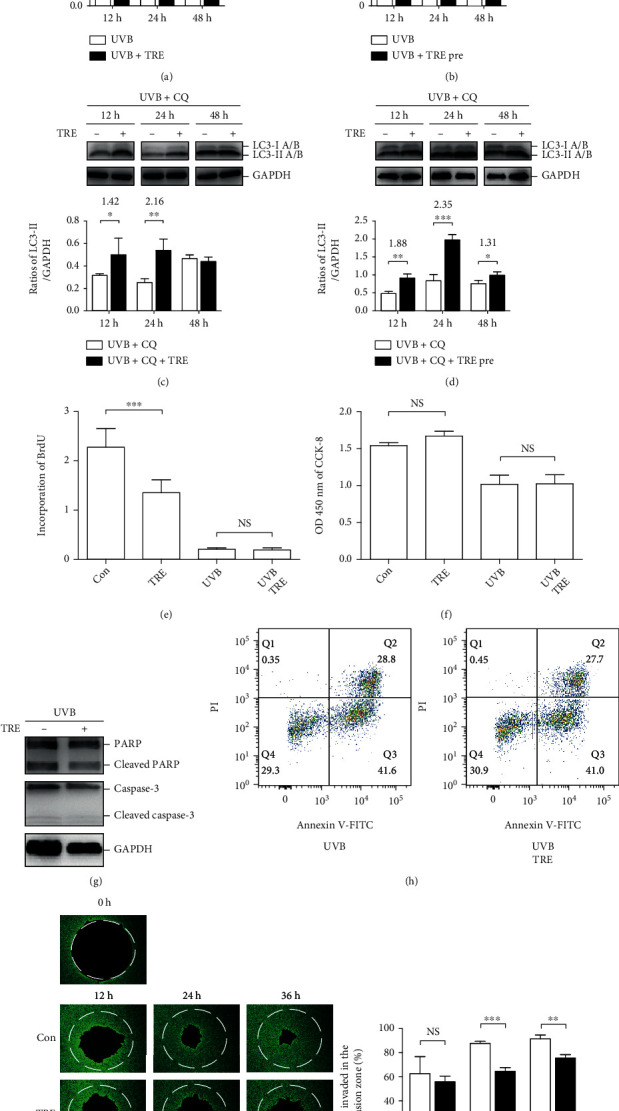
Pretreatment of trehalose induces autophagy and inhibits migration of HaCaT cells. Western blot showing LC3A/B levels in 50 mJ/cm^2^ UVB-irradiated HaCaT cells treated (a, c) or pretreated (b, d) with or without 100 mM trehalose for 12, 24, or 48 hours in the absence (a, b) or presence (c, d) of 50 *μ*M chloroquine. The statistical differences and fold increases of LC3-II/GAPDH ratios were calculated between cells treated with and without trehalose. Afterwards, HaCaT cells were pretreated with or without 100 mM trehalose for 24 hours before exposure to 50 mJ/cm^2^ UVB radiation or not and incubated for 12 hours. (e, f) BrdU and CCK-8 results showing cell proliferation ability. (g) Representative pictures of western blot showing PARP/cleaved PARP and caspase-3/cleaved caspase-3 expression levels. (h) Representative dot plot graphs generated from flow cytometric analysis showing the percentage of apoptotic cells. (i) Representative pictures of calcein AM staining of HaCaT cells in the Cell Migration Assay plate, and the original magnification is ×10. The percentage of cells invaded in the invasion zone was calculated to represent the cell migration rate. ^∗∗∗^*P* ≤ 0.001, ^∗∗^*P* ≤ 0.01, and ^∗^*P* ≤ 0.05. Con: control; h: hour (s); *Q*: quadrant; NS: no significance; Pre: pretreatment; TRE: trehalose.

In this study, to monitor autophagy levels, we examined levels of LC3A/B by western blot and calculated the ratios of LC3-II/GAPDH. First, HaCaT cells were treated with 100 mM trehalose for 12, 24, or 48 hours after 50 mJ/cm^2^ UVB radiation. LC3-II levels increased 2.19-fold in cells treated with trehalose after UVB radiation for 12 hours (*P* < 0.01) (Figure 1(a)). Second, HaCaT cells were pretreated with 100 mM trehalose for 12, 24, or 48 hours before exposure to 50 mJ/cm^2^ UVB radiation. LC3-II levels were increased 3.89-, 4.71-, and 6.53-fold in cells pretreated with trehalose before receiving UVB radiation for 12 (*P* < 0.01), 24 (*P* < 0.001), and 48 (*P* < 0.05) hours, respectively (Figure 1(b)). The increased LC3-II accumulation was markedly more significant in cells pretreated with trehalose. Furthermore, we used chloroquine, a lysosomal inhibitor, to block LC3-II turnover to measure autophagosome formation levels. Increased LC3-II accumulation was observed in HaCaT cells treated with trehalose for 12 (*P* < 0.05) and 24 (*P* < 0.01) hours after UVB radiation, and the fold increases were 1.42 and 2.16, respectively (Figure 1(c)). In cells pretreated with trehalose for 12 (*P* < 0.01), 24 (*P* < 0.001), and 48 (*P* < 0.05) hours, LC3-II accumulation was increased 1.88-, 2.35-, and 1.31-fold, respectively (Figure 1(d)). These results indicate that the induction effect on LC3-II accumulation was more significant in cells pretreated with trehalose for 24 hours.

Following this observation, HaCaT cells were all pretreated with 100 mM trehalose for 24 hours before exposure to 50 mJ/cm^2^ UVB radiation. Cell proliferation was examined using BrdU and CCK-8 assays, and apoptosis levels were assessed by measuring PARP and caspase-3 cleavage and FITC-Annexin V PI staining. The BrdU results showed that trehalose impaired DNA synthesis in normal HaCaT cells (*P* < 0.001) and, unsurprisingly, did not restore the severely impaired DNA synthesis induced by UVB radiation (*P* > 0.05) (Figure 1(e)). The results of the CCK-8 assay showed that trehalose treatment slightly promoted cell proliferation (*P* > 0.05), but it did not exert any effect on UVB-inhibited cell proliferation, in line with the results of the BrdU assay (*P* > 0.05) (Figure 1(f)). We previously reported that UVB radiation triggers apoptosis, which is not affected by postexposure treatment with trehalose [8]. Here, we validated that trehalose pretreatment did not affect PARP or caspase-3 cleavage (Figure 1(g)). The flow cytometry results were in line with the western blot results, which showed that the percentages of apoptotic cells were not altered by trehalose treatment in UVB-irradiated cells (Figure 1(h)).

UVB radiation (50 mJ/cm^2^) has been previously demonstrated to destroy the colony formation ability of normal keratinocytes; thus, in HaCaT cells, we only evaluated the effect of trehalose on cell migration away from UVB radiation [8]. HaCaT cells stained with calcein AM populating the circular void space were observed to monitor cell migration. We found that after incubation with 100 mM trehalose for 24 (*P* < 0.001) and 36 (*P* < 0.01) hours, cells in the invasion zone were decreased, suggesting that cell migration was inhibited (Figure 1(i)).

### 3.2. Trehalose Promotes the Interaction between TIMP3 and Beclin1

**Figure 2:**
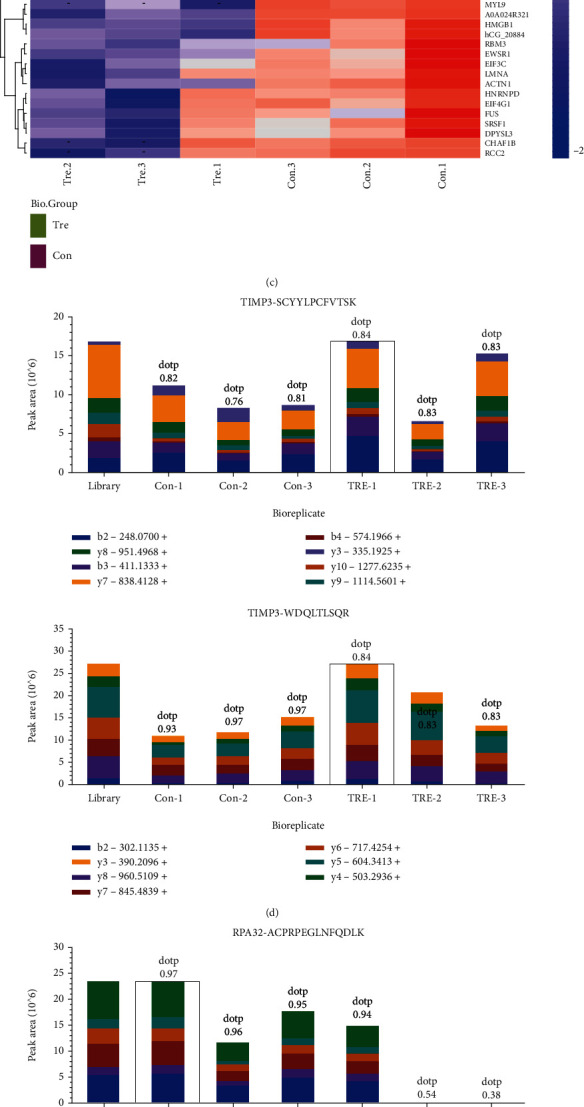
Trehalose promotes the interaction between TIMP3 and Beclin1. (a) HaCaT cells were treated with or without 100 mM or 200 mM trehalose for 12 or 24 hours, respectively. The cell lysate of HaCaT cells was immunoprecipitated with Beclin1 antibody, and levels of ATG14, Rubicon, PI3KC3, PIK3R4, UVRAG, Bcl2, and Beclin1 in the immunoprecipitates and whole cell lysate were analyzed by western blot. In following experiments, HaCaT cells were all treated with trehalose at the concentration of 100 mM for 24 hours. (b) Scatter plot showing global protein expression changes. Orange dots indicate upregulated proteins and green dots indicate downregulated proteins (fold change > 1.5 and *P* < 0.05). (c) The clustering results show the protein expression profiles and patterns of 63 differentially expressed proteins. *Z*-score indicates the relative expression levels of proteins, and the scale of relative expression levels is shown by the color bars, from blue (low) to red (high). (d, e) The absolute quantification of TIMP3 and RPA32 was detected by PRM. (f) Western blot results showing TIMP3, RPA32, and Beclin1 expression levels in the immunoprecipitates and whole cell lysate. (g) Western blot results showing phosphorylation levels of proteins in DNA damage pathway. (h) HaCaT cells were pretreated with or without 100 mM trehalose for 24 hours before exposure to 50 mJ/cm^2^ UVB radiation. Western blot results showing expression levels of TIMP3, RPA32, and Beclin1 in the immunoprecipitates and whole cell lysate. ^∗∗∗^*P* ≤ 0.001, ^∗∗^*P* ≤ 0.01, and ^∗^*P* ≤ 0.05. Con: control; h: hour (s); TRE: trehalose; WCL: whole cell lysate.

Trehalose has been demonstrated to increase autophagy in an mTOR-independent manner, inducing no change in the activities of mTORC1 and mTORC2 or the phosphorylation and expression levels of ATG proteins; therefore, the Beclin1 complex was proposed for possible involvement in autophagy regulation in this setting [8]. As a first effort toward investigating the effect of trehalose on the Beclin1 complex, HaCaT cells were treated with 100 or 200 mM trehalose for 12 and 24 hours, and the levels of ATG14 and RUN domain protein as Bec1 interacting and cysteine-rich containing (Rubicon), PI3KC3, PI3K regulatory subunit (PIK3R) 4, UV radiation resistance-associated gene (UVRAG), and B-cell lymphoma 2 (Bcl2) combined with Beclin1 were determined using coIP and western blot. The results of co-IP and western blotting showed that levels of ATG14, Rubicon, PI3KC3, PIK3R4, UVRAG, and Bcl2 combined with Beclin1 were not affected by trehalose. Expression levels of these proteins in whole-cell lysates were also not altered after exposure to 100 mM trehalose treatment for 24 hours (Figure 2(a)). Second, a protein profile was established to determine the proteins interacting with Beclin1 by label-free quantitative proteomic analysis. By incubating HaCaT cell extracts with the Beclin1 antibody, we enriched a number of its potential binding proteins, 767 of which were identified. After trehalose treatment, 63 differentially binding proteins were identified, including 16 upregulated proteins and 47 downregulated proteins (fold change > 1.5 and *P* < 0.05; Figure 2(b)). Details regarding altered binding proteins are provided in heatmaps (Figure 2(c)). Furthermore, 50 different binding proteins were identified by PRM absolute quantification, and TIMP3 (Figure 2(d)) and RPA32 (Figure 2(e)) were shown to be differentially enriched after trehalose treatment.

Western blot results confirmed the increased binding between TIMP3 and Beclin1 after trehalose treatment (*P* < 0.05) (Figure 2(f)). RPA32, a subunit of RPA that is activated by ATR leading to Chk1 and replication arrest, is important in the regulation of DNA damage signaling [18]. RPA32 was downregulated as shown by PRM absolute quantification, but this result was not confirmed by western blot (Figure 2(f)). In addition to RPA32, phosphorylation of ATR, ATM, Chk1, and histone H2A.X in the DNA damage pathway was not altered by trehalose (Figure 2(g)). Under UVB exposure conditions, even though trehalose significantly decreased the total expression of TIMP3, as shown by whole cell lysate (*P* < 0.01), TIMP3 recruitment to Beclin1 was increased (*P* < 0.001) (Figure 2(h)).

### 3.3. Trehalose Promotes Translocation of ATG9A from the ER to Lysosomes

ATGs have diverse physiologically important roles in membrane trafficking and signaling pathways. ATG9 is the only transmembrane protein of the core autophagy machinery and is proposed to mediate membrane transport to generate autophagosomes [19]. The membrane-bending properties of ATG9 explain its localization to highly curved membranes in cells [20]. ATG9A is located dynamically among organelles, including the Golgi apparatus and endosomes [19]. Furthermore, ER-ATG9A and mitochondria-ATG9A membrane contacts have also been proposed [21, 22].

**Figure 3:**
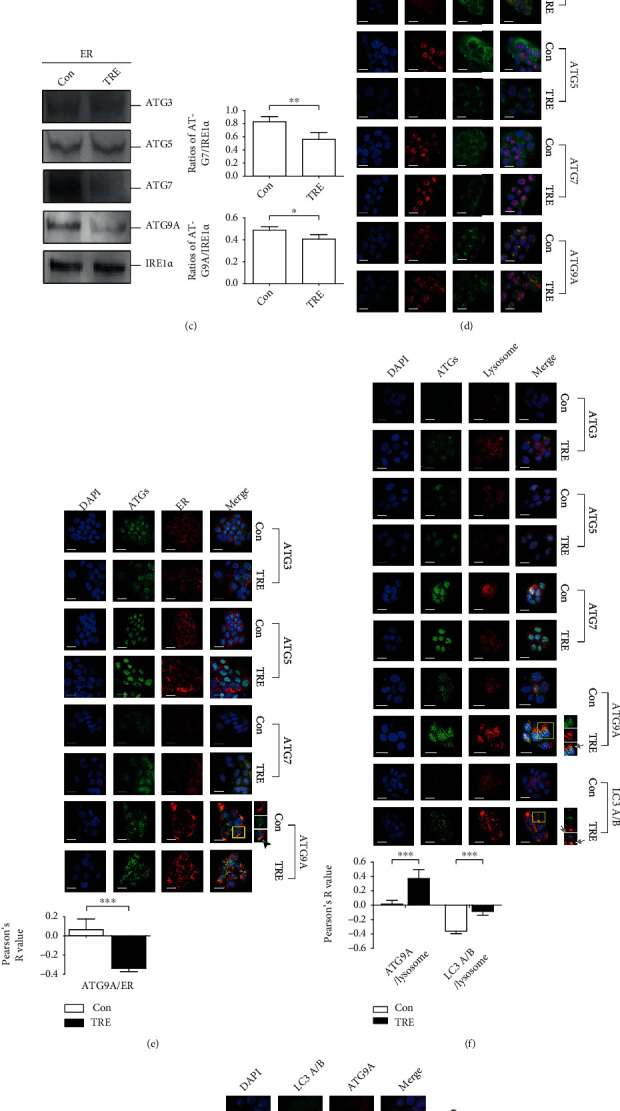
Trehalose promoted the translocation of ATG9A from the ER to lysosomes. HaCaT cells were treated with or without 100 mM trehalose for 24 hours. Representative pictures of western blot showing expression levels of ATG3, ATG5, ATG7, and ATG9A in mitochondria (a), nucleus (b), ER (c), and cytoplasm (a, b). COX IV, IRE1*α*, histone H3, or GAPDH, served as loading controls. The statistical differences of ATG7 and ATG9A levels in the ER were calculated between cells treated with and without trehalose (c). Representative images of IF staining showing the localization of ATGs in mitochondria (ATG3, ATG5, ATG7, and ATG9A) (d), ER (ATG3, ATG5, ATG7, and ATG9A) (e) and lysosomes (ATG3, ATG5, ATG7, ATG9A, and LC3A/B) (f). (g) Representative images of IF staining showing the colocalization of ATG9A and LC3A/B. Scale bar, 20 *μ*m. Enlarged images of colocolization positive cells were included. The statistical differences of Pearson's *R* value representing the localization of ATG9A in ER, ATG9A in lysosome and LC3A/B in lysosome, and the colocolization of ATG9A and LC3A/B was calculated between cells treated with and without trehalose. ^∗∗∗^*P* ≤ 0.001, ^∗∗^*P* ≤ 0.01, and ^∗^*P* ≤ 0.05. Con: control; TRE: trehalose.

We previously demonstrated that trehalose does not affect the total protein expression of ATGs, including ATG3, ATG5, ATG7, and ATG9A [8]. Here, the protein levels of these ATG proteins in the mitochondria, nucleus, ER, and cytoplasm were determined by western blotting. The results showed that the protein levels of ATG3, ATG5, ATG7, and ATG9A in the mitochondria and cytoplasm were not markedly changed by 100 mM trehalose treatment (Figure 3(a)) nor were protein levels in the nucleus (Figure 3(b)), indicating that trehalose did not affect the localization of these ATGs in the mitochondria, nucleus, or cytoplasm. However, protein levels of ATG7 (*P* < 0.01) and ATG9A (*P* < 0.05) in the ER were decreased after trehalose treatment (Figure 3(c)). Subsequently, we examined the localization of ATG3, ATG5, ATG7, and ATG9A in different organelles, including the mitochondria, ER, and lysosomes, using IF staining. The confocal results showed that the localization of ATGs in mitochondria and ATG3, ATG5, and ATG7 in the ER were not changed after trehalose treatment (Figures 3(d) and 3(e)). Similar to the western blot results, ATG9A displayed decreased localization in the ER (*P* < 0.001) (Figure 3(e)). With regard to the important role of core ATGs, especially LC3, in autophagosome-lysosome fusion, we examined the localization of ATGs and LC3 in lysosomes. The localization of ATG3, ATG5, and ATG7 in lysosomes was not changed after trehalose treatment, while ATG9A (*P* < 0.001) and LC3 (*P* < 0.001) displayed increased localization (Figure 3(f)). Furthermore, we costained ATG9A and LC3 to examine the colocalization of these two proteins. ATG9A exhibited increased colocalization with LC3 after trehalose treatment (*P* < 0.001) (Figure 3(g)). These results indicate increased translocation of ATG9A from the ER to lysosomes in response to trehalose treatment.

### 3.4. TIMP3 and ATG9A Contribute to Trehalose-Induced Autophagy and Trehalose-Inhibited Cell Death in UVB-Irradiated HaCaT Cells

**Figure 4:**
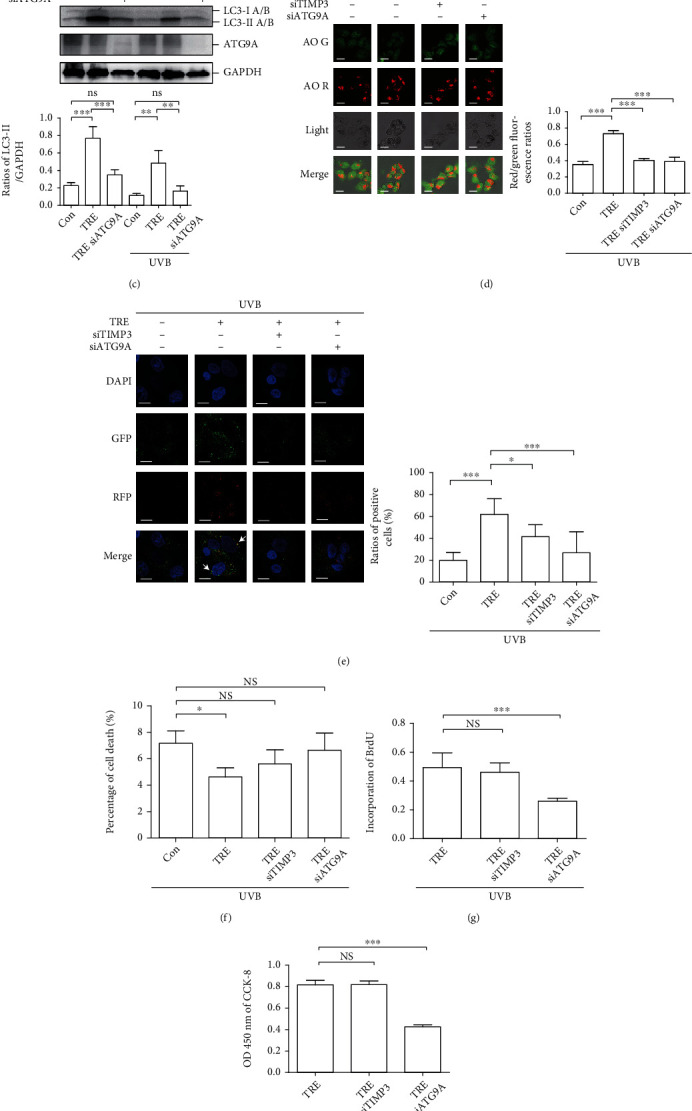
TIMP3 and ATG9A contribute to trehalose-induced autophagy and cell death in UVB-irradiated HaCaT cells. (a) Western blot results showing TIMP3 and ATG9A expression levels in siRNA transfected HaCaT cells. HaCaT cells transfected by TIMP3/ATG9A siRNA or not were pretreated with 100 mM trehalose for 24 hours before exposure to 50 mJ/cm^2^ UVB radiation and incubated for 12 hours. (b) Western blot results showing LC3A/B and MMP9 expression levels. Ratios of LC3-II/GAPDH and MMP9/GAPDH were calculated. (c) Western blot results showing LC3A/B levels. Ratios of LC3-II/GAPDH were calculated. (d) Representative images of AO staining. Scale bar, 20 *μ*m. Red/green fluorescence ratios were calculated to display autophagy levels. (e) Representative images of RFP-GFP-LC3B puncta analysis. Scale bar, 20 *μ*m. The ratios of positive cells with yellow fluorescence were calculated to display autophagy levels. (f) LDH results showing the percentage of cell death. (g) BrdU results showing DNA synthesis levels. (h) CCK-8 results showing cell proliferation capacity. (i) Representative pictures of calcein AM staining of HaCaT cells in the Cell Migration Assay plate, and the original magnification is ×10. The percentage of cells invaded in the invasion zone was calculated to represent the cell migration rate. ^∗∗∗^*P* ≤ 0.001, ^∗∗^*P* ≤ 0.01, and ^∗^*P* ≤ 0.05. Con: control; h: hour (s); NS: no significance; TRE: trehalose.

HaCaT cells were transfected with TIMP3, ATG9A, or nontargeting siRNA, and protein expression levels of TIMP3 and ATG9A were confirmed to be downregulated by siRNA treatment (Figure 4(a)). Then, the role of TIMP3 and ATG9A in autophagy was evaluated by determining LC3-II levels and red/green fluorescence ratios in AO-stained cells. An impact of TIMP3 siRNA on LC3-II levels was not obvious (*P* > 0.05, TRE + siTIMP3 vs. TRE group; *P* > 0.05, UVB + TRE + siTIMP3 vs. UVB + TRE group) (Figure 4(b)). Of note, levels of matrix metallopeptidase (MMP) 9, a substrate of TIMP3, were upregulated in response to TIMP3 siRNA in normal trehalose-treated cells (*P* < 0.05 vs. TRE group), which was not changed in cells exposed to UVB radiation (*P* > 0.05, UVB + TRE + siTIMP3 vs. UVB + TRE group) (Figure 4(b)). This result could be explained by the phenomenon that increased.

TIMP3 was recruited to Beclin1 under UVB exposure conditions, which is shown in Figure 2(h). In HaCaT cells away from UVB radiation, ATG9A siRNA neutralized the trehalose-increased LC3-II levels (*P* > 0.05 vs. Con group). Similarly, in cells exposed to UVB radiation, ATG9A siRNA not only decreased but also reversed LC3-II levels (*P* < 0.01 vs. UVB + TRE group; *P* > 0.05 vs. UVB group) (Figure 4(c)). In AO-stained cells, the nuclei and cytoplasm displayed deep green and slight green fluorescence, respectively, whereas the acidic vesicular organelles displayed red fluorescence. Here, the ratios of red/green fluorescence (adjusted volume) were calculated to monitor autophagy levels. Unlike the western blot results, TIMP3 and ATG9A siRNA both downregulated the ratios of red/green fluorescence compared to the TRE group in UVB-irradiated cells (*P* < 0.001) (Figure 4(d)). The tandem sensor RFP-GFP-LC3B was subsequently used to monitor autophagic induction. Autophagy levels were assessed by measuring the ratios of positive cells with yellow puncta. Consistent with the AO staining results, TIMP3 (*P* < 0.05) and ATG9A (*P* < 0.001) siRNA downregulated the ratios of positive cells compared to the TRE group in UVB-irradiated cells (Figure 4(e)).

Afterward, the roles of TIMP3 and ATG9A in cell death, DNA synthesis, cell proliferation, and migration were evaluated. In our previous study, we demonstrated that trehalose attenuated UVB-induced LDH release [8]. Here, the LDH assay data revealed that TIMP3 and ATG9A siRNA reversed trehalose-inhibited cell death in UVB exposure conditions (*P* < 0.05, UVB + TRE vs. UVB group; *P* > 0.05, UVB + TRE + siTIMP3/siATG9A vs. UVB group) (Figure 4(f)). The BrdU assay data showed that TIMP3 siRNA did not increase the incorporation (*P* > 0.05), and ATG9A siRNA even downregulated the incorporation (*P* < 0.001) compared to the UVB + TRE group (Figure 4(g)). The CCK-8 assay data coincided with the BrdU result that TIMP3 siRNA did not increase the absorbance (*P* > 0.05), and ATG9A siRNA downregulated the absorbance at 450 nm (*P* < 0.001) compared to the UVB + TRE group (Figure 4(h)). Finally, the migration measurement was performed. Using ATG9A siRNA, the area without cell migration was even larger than that in the original group (0 hour), indicating that ATG9A accounts for cell survival; thus, statistical analysis of the siATG9A and siATG9A + TRE groups was not performed. The results showed that cells in the invasion zone were decreased in response to trehalose treatment (*P* < 0.01 TRE vs. Con group) and did not display a significant difference between the TRE group and the siTIMP3 + TRE group, indicating that TIMP3 siRNA does not attenuate trehalose-inhibited cell migration (*P* > 0.05) (Figure 4(i)).

### 3.5. TIMP3 and ATG9A Contribute to Trehalose-Induced Autophagy and Cell Death and Trehalose-Inhibited Migration of A431 Cells

**Figure 5:**
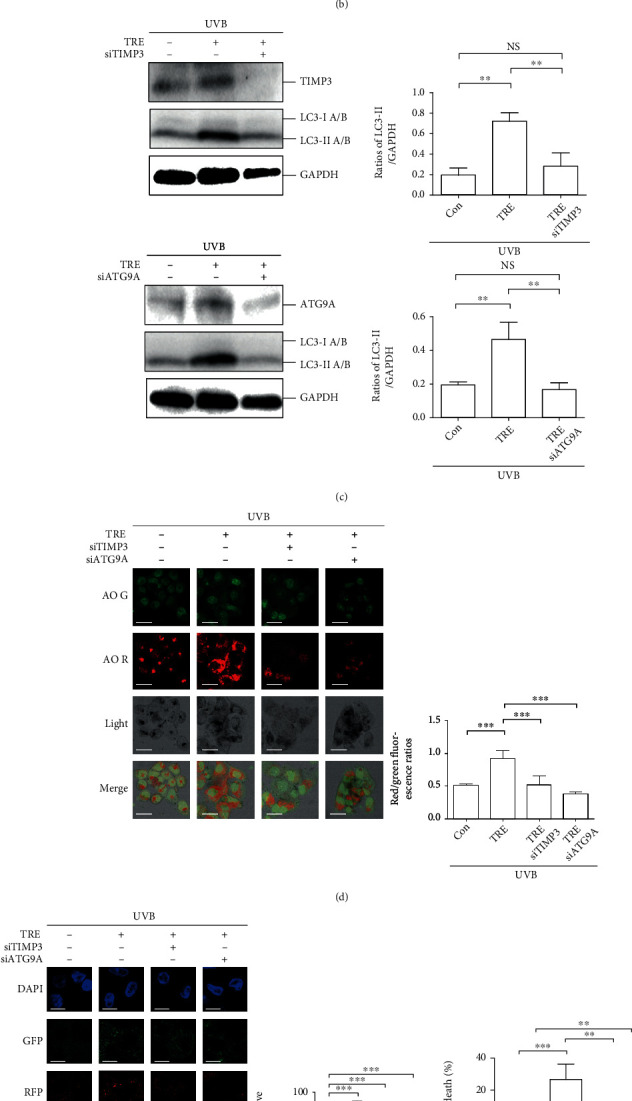
TIMP3 and ATG9A contribute to mediating the impact of trehalose on autophagy, cell death, and migration of A431 cells. (a) The mRNA levels of TIMP3 and ATG9A detected by RT-qPCR in normal (*n* = 8), AK (*n* = 8), and cSCC (*n* = 8) skin tissues. U6 served as the endogenous control. (b) Representative pictures of IHC staining of TIMP3 and ATG9A in normal, AK, and cSCC skin tissues. Scale bar, 100 *μ*m. Enlarged images were included. The integrated optical density of TIMP3 and ATG9A of normal (*n* = 8), AK (*n* = 8), and cSCC (*n* = 8) skin tissue slides were obtained from eight visions of four slides. In following experiments, A431 cells were pretreated with 100 mM trehalose for 24 hours before exposure to 50 mJ/cm^2^ UVB radiation and then incubated for 4 hours. (c) Western blot results showing TIMP3, ATG9A, and LC3A/B expression levels. Ratios of LC3-II/GAPDH were calculated. (d) Representative images of AO staining. Red/green fluorescence ratios were calculated to display autophagy levels. Scale bar, 20 *μ*m. (e) Representative images of RFP-GFP-LC3B punta analysis. Scale bar, 20 *μ*m. The ratios of positive cells with yellow fluorescence was calculated to display autophagy levels. (f) LDH results showing the percentage of cell death. (g) Representative pictures of calcein AM staining of A431 cells in the Cell Migration Assay plate, and the original magnification is ×10. The percentage of cells invaded in the invasion zone was calculated to represent the cell migration rate. ^∗∗∗^*P* ≤ 0.001, ^∗∗^*P* ≤ 0.01, and ^∗^*P* ≤ 0.05. Con: control; h: hour (s); IOD: integrated optical density; NS: no significance; TRE: trehalose.

To determine the expression pattern of TIMP3 and ATG9A in UVB radiation-induced skin diseases, we assessed mRNA levels of TIMP3 and ATG9A in normal, AK, and cSCC skin tissues. The mean age of the entire sample was 71.2 years with an SD of 12.9, with 11 males and 15 females. Normal skin samples serving as controls were obtained from the abdomen, thigh, and upper arm with a very low level of solar UV exposure. AK and cSCC samples were all from facial skin with a high level of solar UV exposure. The data showed that compared to normal skin tissues, mRNA levels of TIMP3 and ATG9A were not markedly changed in AK skin tissues (*P* > 0.05). In contrast, compared to normal or AK skin samples, mRNA levels of TIMP3 (*P* < 0.05) and ATG9A (*P* < 0.01) were significantly upregulated in cSCC skin tissue samples (Figure 5(a)). This result prompted us to further examine protein levels and presence in skin tissues by IHC analysis. The mean age for the entire sample was 65.4 years with an SD of 15.9, with 5 males and 7 females. As shown, compared to the normal skin, overall TIMP3 protein levels were upregulated in both AK (*P* < 0.05) and cSCC (*P* < 0.05) skin tissues. The overall ATG9A protein levels were upregulated in AK (*P* < 0.05) but not in cSCC (*P* > 0.05) skin tissues (Figure 5(b)). Despite the differential performance of TIMP3 and ATG9A in the mRNA and IHC results, the results above suggest aberrant upregulation of TIMP3 and ATG9A in AK and cSCC tissues.

Based on this observation, TIMP3 and ATG9A were next downregulated using siRNAs in A431 cells to investigate the role of the two genes in autophagy, cell death, and migration in cutaneous tumor cells. We found that A431 cells were unexpectedly sensitive to UVB radiation. After incubation for 12 hours post-UVB radiation, more than 50% of the cells were dead and suspended. Thereafter, A431 cells were incubated for only 4 hours after 50 mJ/cm^2^ UVB exposure. As shown in A431 cells exposed to UVB radiation, trehalose treatment increased LC3-II levels (*P* < 0.01 vs. UVB group), while TIMP3 (*P* < 0.01 vs. UVB + TRE group) and ATG9A (*P* < 0.01 vs. UVB + TRE group) siRNA both decreased and even reversed (*P* > 0.05 vs. UVB group) trehalose-increased LC3-II levels (Figure 5(c)). The results of AO staining were in line with the western blot results. The ratios of red/green fluorescence upregulated by trehalose were attenuated by TIMP3 and ATG9A siRNA (*P* < 0.001) (Figure 5(d)). Consistent with the AO staining results, TIMP3 (*P* < 0.001) and ATG9A (*P* < 0.001) siRNA both downregulated the ratios of positive cells compared to the TRE group in UVB-irradiated cells (Figure 5(e)). As shown in the LDH assay, UVB radiation decreased the percentage of cell death to a minimum, which is opposite to the results in HaCaT cells. Trehalose significantly increased cell death (*P* < 0.001 vs. UVB group), exhibiting a clearance effect on tumor cells, and TIMP3 and ATG9A siRNA inhibited trehalose-induced cell death under UVB exposure conditions (*P* < 0.01 vs. UVB + TRE group) (Figure 5(f)). Trehalose also inhibited the migration of A431 cells, as the number of cells in the invasion zone was decreased compared to that in the control group (*P* < 0.001). Both TIMP3 and ATG9A siRNA increased cell invasion in the invasion zone (*P* < 0.05 vs. the TRE group) (Figure 5(g)). Overall, these results revealed that the effect of trehalose on cell death and migration is impaired by neutralizing TIMP3 or ATG9A siRNA, suggesting the involvement of both genes in promoting cell death and inhibiting migration of A431 cells.

### 3.6. ATG9A Contributes to Trehalose-Induced Autophagy and Trehalose-Inhibited IL8 Expression in HEKs

**Figure 6:**
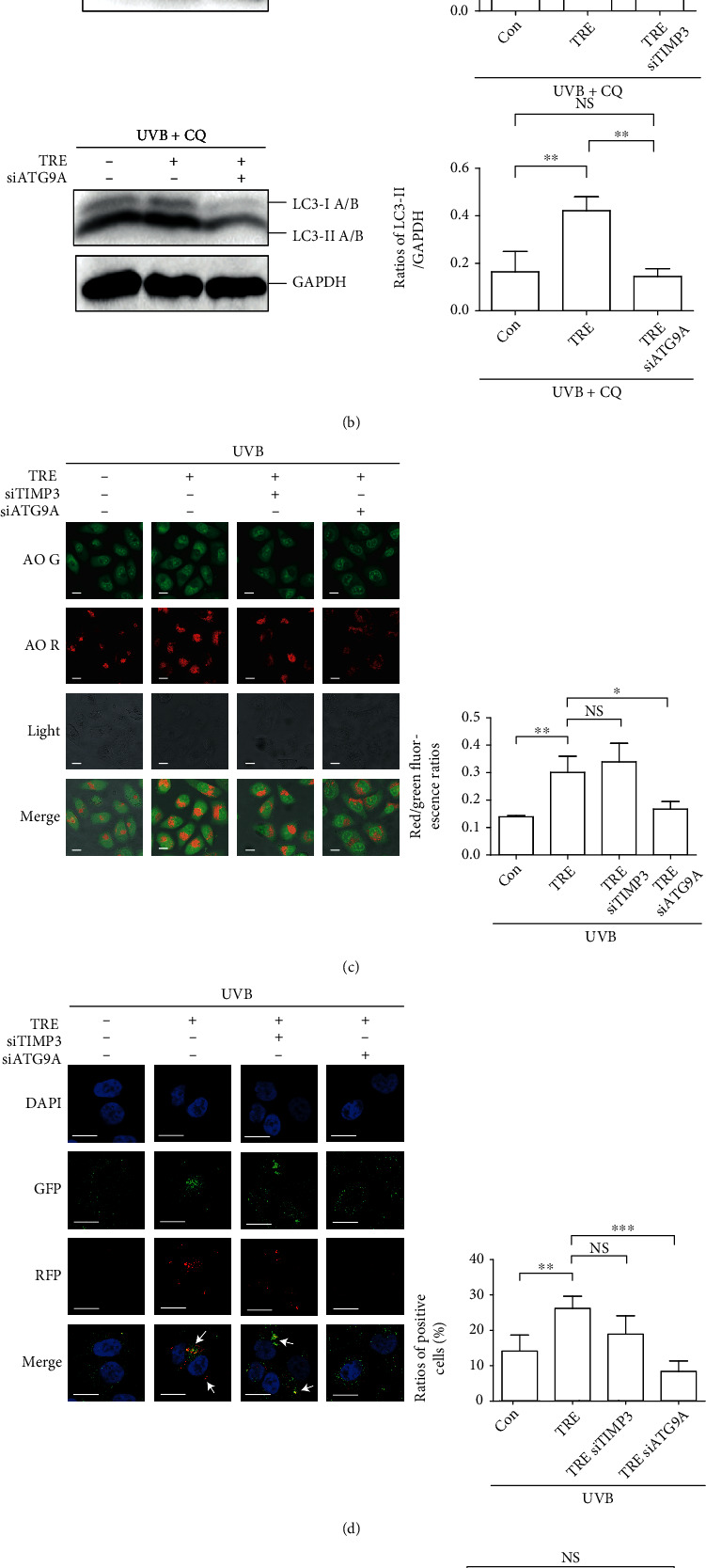
The contribution of TIMP3 and ATG9A in mediating the impact of trehalose on autophagy, cell death, and IL8 expression in HEKs. HEKs transfected by TIMP3/ATG9A siRNA or not were pretreated with 100 mM trehalose for 24 hours before exposure to 50 mJ/cm^2^ UVB radiation and incubated for 12 hours. (a) Western blot results showing TIMP3, ATG9A, and LC3A/B expression levels. (b) Western blot results showing LC3A/B levels in the presence of chloroquine. Ratios of LC3-II/GAPDH were calculated (a, b). (c) Representative images of AO staining. Scale bar, 20 *μ*m. Red/green fluorescence ratios were calculated to display autophagy levels. (d) Representative images of RFP-GFP-LC3B punta analysis. Scale bar, 20 *μ*m. The ratios of positive cells with yellow fluorescence were calculated to display autophagy levels. (e) LDH results showing the percentage of cell death. (f) RT-qPCR results showing IL8 mRNA levels. (g) ELISA results showing levels of IL8 secreted to the culture medium. ^∗∗∗^*P* ≤ 0.001, ^∗∗^*P* ≤ 0.01, and ^∗^*P* ≤ 0.05. Con: control; CQ: chloroquine; NS: no significance; TRE: trehalose.

Because we have demonstrated that TIMP3 and ATG9A possibly play an inhibitory role in the survival and migration of A431 cells, we were curious about the behavior of TIMP3 and ATG9A in normal keratinocytes. To determine whether TIMP3 and ATG9A are involved in autophagy, cell death, and inflammatory regulation in normal keratinocytes, both genes were downregulated using siRNAs in HEKs. In cells exposed to UVB radiation, TIMP3 and ATG9A siRNA did not attenuate the increased LC3-II level induced by trehalose (*P* > 0.05), which is distinct from the data obtained in HaCaT and A431 cells (Figure 6(a)). Accordingly, chloroquine was used to block LC3-II turnover to measure autophagosome formation. As shown, ATG9A siRNA reversed trehalose-induced LC3-II accumulation (*P* < 0.01 vs. UVB + TRE group; *P* > 0.05 vs. UVB group), while TIMP3 siRNA did not exert any effect (*P* > 0.05 vs. UVB + TRE group) (Figure 6(b)). The results of AO staining and RFP-GFP-LC3B puncta analysis conformed to the western blot results. The ratios of red/green fluorescence upregulated by trehalose were attenuated by ATG9A siRNA (*P* < 0.01) but not TIMP3 siRNA (*P* > 0.05) (Figure 6(c)). The ratios of positive cells with yellow puncta fluorescence upregulated by trehalose were attenuated by ATG9A siRNA (*P* < 0.001) but not TIMP3 siRNA (*P* > 0.05) (Figure 6(d)). The inhibitory effect of trehalose on cell death was impaired by TIMP3 and ATG9A siRNA under UVB exposure conditions; however, only TIMP3 siRNA reversed the effect of trehalose on cell death (*P* < 0.05 vs. the UVB + TRE group; *P* > 0.05 vs. the UVB group), and ATG9A siRNA slightly promoted cell death (*P* > 0.05 vs. the UVB + TRE group) (Figure 6(e)). In a previously published cytokine array assay dataset, UVB radiation induced IL8 expression in HEKs [23]. Here, the levels of IL8 mRNA and protein secreted into the culture medium by HEKs exposed to UVB and treated with trehalose or siRNA were next determined. Trehalose downregulated IL8 mRNA levels in UVB-irradiated HEKs (*P* < 0.001 vs. UVB group), ATG9A siRNA reversed trehalose-inhibited IL8 mRNA expression (*P* < 0.01 vs. UVB + TRE group; *P* > 0.05 vs. UVB group), and TIMP3 siRNA did not exert any effect (*P* > 0.05 vs. UVB + TRE group) (Figure 6(f)). The ELISA results were in accordance with the mRNA quantification. Trehalose downregulated IL8 levels in UVB-irradiated HEKs (*P* < 0.01 vs. UVB group), ATG9A siRNA reversed trehalose-inhibited IL8 expression (*P* < 0.05 vs. UVB + TRE group; *P* > 0.05 vs. UVB group), and TIMP3 siRNA did not exert any effect (*P* > 0.05 vs. UVB + TRE group) (Figure 6(g)). Taken together, in HEKs, the ATG9A gene mediates the effect of trehalose on autophagy and IL8 expression, while TIMP3 contributes to the inhibitory effect of trehalose on cell death, irrespective of autophagy regulation.

## 4. Discussion

Many studies have reported that trehalose induces autophagy in an mTOR-independent manner, but the related molecular mechanism is not well understood. Here, we sought to identify essential molecules in the autophagy machinery that are regulated by trehalose. In this study, two molecules, TIMP3 and ATG9A, were demonstrated to be involved in mTOR-independent autophagy regulation. We found that the interaction between TIMP3 and Beclin1 and the translocation of ATG9A from the ER to lysosomes might contribute to mediating trehalose-induced autophagy in keratinocytes. Then, we focused on whether TIMP3 and ATG9A contribute to the cytoprotective effect of trehalose, discovering that either TIMP3 or ATG9A siRNA impaired the effect of trehalose on cell death, migration, or inflammation. In addition, AK and cSCC tissues exhibited aberrant upregulation of TIMP3 and ATG9A, suggesting the potential application of trehalose in the treatment of related skin diseases by targeting TIMP3 or ATG9A.

Beclin1, the mammalian ortholog of yeast Atg6, is considered to play a central role in incipient autophagy due to its interaction with other molecules. The classical Beclin1 complex is composed of ATG14, PI3KC3, and PIK3R4 [24]. Subsequently, numerous proteins that interact with Beclin1 were identified [25]. In this study, we determined the levels of ATG14, Rubicon, PI3KC3, PIK3R4, UVRAG, and Bcl2 combined with Beclin1 in the absence and presence of trehalose. Surprisingly, the interactions between these molecules and Beclin1 were found to be not altered by trehalose (Figure 2(a)). Accordingly, the global proteins interacting with Beclin1 were identified, and differentially expressed proteins were explored (Figures 2(b) and 2(c)). Our study demonstrates a previously unknown mechanism by which TIMP3, an MMP inhibitor, interacts with Beclin1 and is promoted by trehalose. In the past, the involvement of TIMP3 in autophagy regulation was mentioned, but the related mechanism was not defined in detail. MMP inhibition was reported to be associated with increased levels of autophagy markers [26]. Our investigations revealed the contribution of TIMP3 to autophagy regulation, as evidenced by the inhibition of autophagy in HaCaT (Figures 4(b) and 4(d)) and A431 cells (Figures 5(c) and 5(d)) by TIMP3 siRNA.

Regarding human skin cells, TIMP3 exhibits the rhythmic expression in synchronized human keratinocytes and contributes to epidermal homeostasis. The expression of TIMP3 was downregulated by UVB radiation, and the circadian expression of TIMP3 conversely inhibited UVB-induced inflammation [27, 28]. Nevertheless, in this study, TIMP3 siRNA did not display efficacy in recovering trehalose-inhibited inflammation (IL8 expression) in UVB-irradiated HEKs (Figures 6(e) and 6(f)), which we interpret as the defeated regulation of autophagy under UVB radiation conditions (Figures 6(a)–6(c)); since in our previous study, induced autophagic flux was demonstrated to resist UVB-induced inflammation [23]. We also hypothesized that autophagy modulated by the interaction between TIMP3 and Beclin1 contributes to regulating cell death and migration of keratinocytes, but the related mechanism was not clarified.

ATG9A is one of the least understood components of the autophagic machinery, and thus far, there are few reports describing the behavior of ATG9A in keratinocytes. Our study revealed the involvement of ATG9A in regulating keratinocyte proliferation, death, inflammation, and migration. Most importantly, as we previously demonstrated that trehalose induced autophagy without altering the expression of ATG9A, in this study, we further demonstrated that the translocation of ATG9A among intracellular organelles contributes to the induction effect of trehalose on autophagy. ATG9A is the only transmembrane protein of the core autophagy machinery, and structural analysis explains the localization of ATG9A to small vesicles and highly curved edges of growing autophagosomes [20]. ATG9A was proposed to traffic through multiple organelles, including the Golgi network, endosomes, ER, mitochondria, autophagosomes, and the ATG9 compartment [29–33]. To date, there have been no reports on the localization of ATG9A (or ATG5) to the nucleus, but ATG9A is required for translocation to the nucleus of some master regulators of lysosomal biogenesis [34]. In addition to ATG9A, the localization of many other ATGs in organelles has been documented [35]. Thus, evaluating the influence of trehalose on the intracellular location of ATGs is an important method for exploring the mechanism of its induction effect on autophagy. In this study, we identified the alteration of ATG9A protein expression in the ER by western blotting (Figure 3(c)). Consequently, the IF results showed that localization of ATG9A in the ER was decreased, while localization in lysosomes and colocalization with LC3A/B were increased by trehalose (Figures 3(e)–3(g)). These results raise the intriguing possibility that trehalose promotes the translocation of ATG9A from the ER to lysosomes.

The in vitro data from HaCaT cells demonstrated that TIMP3 and ATG9A mediate the induction effect of trehalose on cell death against UVB radiation. Consequently, we evaluated the levels of TIMP3 and ATG9A in UVB exposure-related epidermal diseases, including AK and cSCC. Our data confirmed the upregulation of TIMP3 in cSCC skin tissues at both the mRNA and protein levels. Regarding ATG9A, even though the aberrant expression in AK and cSCC skin tissues was demonstrated compared to the normal skin, the data from RT–qPCR were not completely in line with the IHC analysis (Figures 5(a) and 5(b)). The involvement of TIMP3 in AK and cSCC is controversial [36, 37].

MMPs are a family of proteolytic enzymes capable of degrading components of the extracellular membrane, and their activities are controlled by TIMPs. Among the four types of TIMPs, TIMP3 has the widest range of MMP inhibition capacity [36]. In this study, the decreased TIMP3 expression by siRNA led to the upregulation of MMP9, but in HaCaT cells exposed to UVB radiation, decreased TIMP3 failed to upregulate MMP9, which was not in agreement with traditional understanding (Figure 4(b)). Interestingly, TIMP3 combined with Beclin1 was increased by trehalose (Figure 2(h)), which we speculated to contribute to failed regulation of the substrate MMP9 under UVB radiation conditions (Figure 4(b)).

In conclusion, we demonstrated that the interaction between TIMP3 and Beclin1 and the translocation of ATG9A from the ER to lysosomes are promoted by trehalose. TIMP3 and ATG9A contribute to mediating the effect of trehalose on autophagy, cell death, and IL8 expression in keratinocytes in response to UVB radiation. These two genes were also revealed to be involved in regulating A431 migration. However, more evidence is required to determine the involvement of TIMP3 and ATG9A in the occurrence or development of UV exposure-related skin diseases because the RT–qPCR results did not entirely agree with the IHC results. Based on its ability to modulate autophagy, trehalose has been considered an innovative drug for ameliorating injured cornea healing, neurodegenerative diseases, cardiac remodeling, metabolic diseases, and viral infection [1, 38–41]. Since TIMP3 and ATG9A were demonstrated to be involved in autophagy regulation by trehalose, our study proposes two molecular targets of trehalose, which is remarkable for defining the molecular mechanism and expanding the application of trehalose. In addition, in-depth understanding of the molecular mechanism of UVB radiation-induced skin diseases has the potential to directly impact therapy and diagnosis.

## Data Availability

All the data supporting the results were shown in the paper can be available from the corresponding author.
